# Cloning and Characterization of a Cold-adapted Chitosanase from Marine Bacterium *Bacillus* sp. BY01

**DOI:** 10.3390/molecules24213915

**Published:** 2019-10-30

**Authors:** Yue Yang, Zhou Zheng, Yifei Xiao, Jiaojiao Zhang, Yu Zhou, Xiao Li, Shangyong Li, Huiqing Yu

**Affiliations:** 1 School of Basic Medicine, Qingdao University, Qingdao 266071, China; yueyueyoung@126.com (Y.Y.); abc1286963266@163.com (Y.X.); 13220886636@163.com (J.Z.); zy18339956716@163.com (Y.Z.); lilix0823@163.com (X.L.); 2 First Institute of Oceanography, Ministry of Natural Resource, Qingdao 266061, China; zhengzhou@fio.org.cn

**Keywords:** chitosanase, *Bacillus* sp. BY01, cold-adaption

## Abstract

Chitosanase plays an important role in the production of chitooligosaccharides (CHOS), which possess various biological activities. Herein, a glycoside hydrolase (GH) family 46 chitosanase-encoding gene, *csnB*, was cloned from marine bacterium *Bacillus* sp. BY01 and heterologously expressed in *Escherichia coli*. The recombinant chitosanase, CsnB, was optimally active at 35 °C and pH 5.0. It was also revealed to be a cold-adapted enzyme, maintaining 39.5% and 40.4% of its maximum activity at 0 and 10 °C, respectively. Meanwhile, CsnB showed wide pH-stability within the range of pH 3.0 to 7.0. Then, an improved reaction condition was built to enhance its thermostability with a final glycerol volume concentration of 20%. Moreover, CsnB was determined to be an endo-type chitosanase, yielding chitosan disaccharides and trisaccharides as the main products. Overall, CsnB provides a new choice for enzymatic CHOS production.

## 1. Introduction

Chitin, the second most important natural polymer compound after cellulose on earth [1], is an insoluble liner polymer of β-1,4-linked *N*-acetyl-d-glucosamine [2,3]. As a totally or partially deacetylated derivative of chitin, chitosan is a water-soluble polysaccharide composed of *N*-acetyl-d-glucosamine (GlcNAc) and d-glucosamine (GlcN) residues [4,5]. Thus far, chitosan has been widely used for ecological, agricultural, food, and biomedical applications, mainly due to its physicochemical and biological properties [4].

Chitosanases (EC.3.2.1.132) are enzymes that catalyze the hydrolysis of the β-1,4 glycosidic bond of chitosan producing chitooligosaccharides (CHOS) [5]. CHOS has been shown to have potential medical functions [6,7], including but not limited to anti-bacterial effect [8], anti-oxidant effect [9], anti-obesity effect [10], hypoglycemic effect [9,11], and anti-tumor effect [7]. These excellent properties are why CHOS is widely used in agriculture, cosmetics, food, medicine, and other fields [6,12,13]. According to the similarity in amino acid sequences, chitosanases were classified into GH families 5, 7, 8, 46, 75, and 80 in the Carbohydrate-Active Enzymes (CAZy) database [14,15,16,17,18]. Families GH5, GH7 and GH8 consist of chitosanases and other glycoside hydrolases. By contrast, families GH46, GH75 and GH80 comprise exclusively of chitosanases [17]. To date, the majority of the reported chitosanases have belonged to GH46. Although hundreds of chitosanases have been purified, cloned, and characterized, enzymes for commercial use are rather rare. Therefore, it is still very urgent to screen and discover new chitosanases, especially enzymes with special properties such as thermo-recovery, thermo-stability, and cold-adaptability. Cold-adapted enzymes can perform good enzymatic activities at low temperatures [19], making them available to save resources and help protect the environment [5]. Reacting at low temperatures can also reduce the possibility of bacterial contamination. Additionally, they are useful for room temperature reactions without requiring heating. However, only few cold-adapted chitosanases have been reported. In addition, the reported cold-adapted chitosanases are often unstable [19] when the reaction temperature slightly increases (more than 30 °C), which limits its application in industrial production.

In the study, a GH46 chitosanase-encoding gene, *csnB*, was cloned from *Bacillus* sp. BY01 and heterologously expressed in *Escherichia coli*. Its expression product CsnB was confirmed to be a cold-adapted chitosanase. In order to overcome the deficiencies in the industrial production process of cold-adapted enzymes, we improved the reaction conditions by means of adding glycerol and exploring its appropriate concentration. The thermo-stability of CsnB was strongly improved when mixed with suitable concentrations of glycerol. Additionally, it was found that CsnB is an endo-type chitonase, resulting in disaccharide and trisaccharide degradation products.

## 2. Results and Discussion

### 2.1. Sequence Analysis of csnB/CsnB

In this study, a sea mud sample from Yellow Sea sediment was spread on selection plates containing chitosan (0.50%) and a chitosanase-producing bacterial strain was isolated. The bacterial colony is white and shiny, with neat edges, a smooth surface, and a protruding center. According to the 16S rRNA gene sequence analysis, the strain was identified and named *Bacillus* sp. BY01. The genome sequence analysis of strain *Bacillus* sp. BY01 showed that it contained a chitosanase coding region, named *csnB*. The gene contains a 900 bp open reading frame (ORF), which encodes a chitosanase, CsnB, consisting of 299 amino acid residues. Signal peptide analysis shows that CsnB contains a putative signal peptide (Met1 to Ala22) in its N-terminus. According to a search of the Conserved Domain Database (CDD) of NCBI, CsnB is presumed to be a chitosanase with a single-domain belonging to the glycosyl hydrolase 46 family. The theoretical isoelectric point (pI) and molecular weight (Mw) of CsnB were 7.65 and 30.89 kDa, respectively.

**Figure 1:**
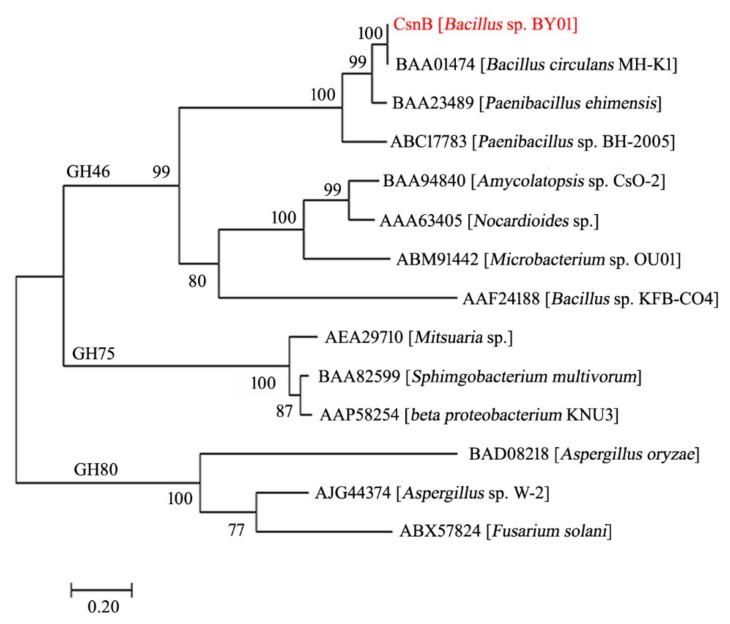
Phylogenetic analysis of CsnB and other chitosanases. The neighbor-joining tree indicates phylogenetic relationships between CsnB and other GH family 46, 75, and 80 members. The scale bar indicates the average number of amino acid substitutions per site. The bootstrap test of the tree was performed with 1000 replications.

**Figure 2:**
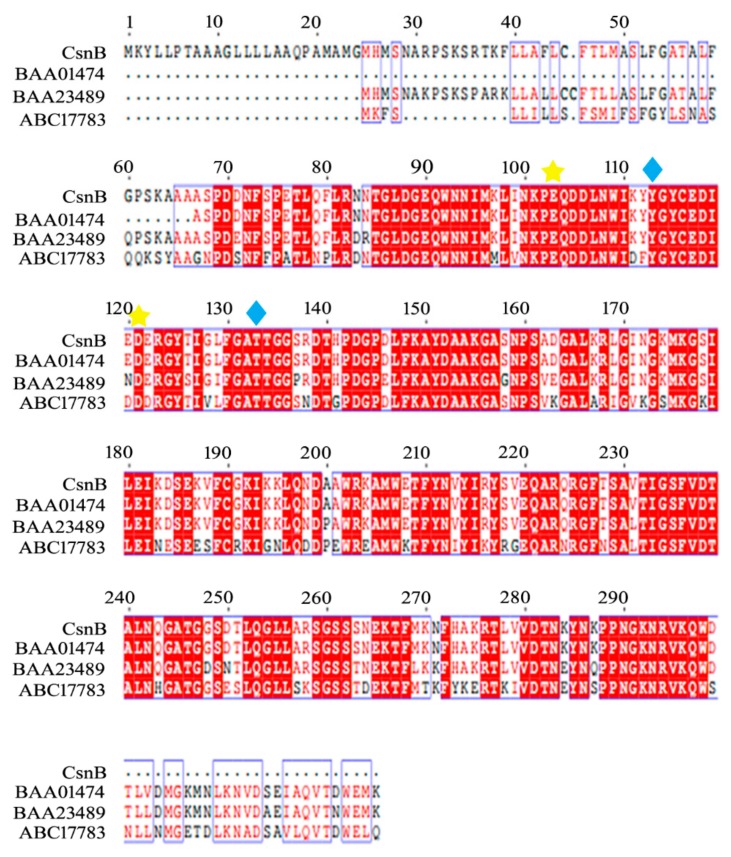
Sequence comparison of CsnB with related chitosanases from GH family 46. Chitosanase MH-Kl (Genbank number: BAA01474); chitosanase from *Paenibacillus ehimensis* (Genbank number: BAA23489) and chitosanase from *Paenibacillus* sp. BH-2005 (Genbank number: ABC17783). The identical substrate binding and catalytic sites were marked with blue rhombus and yellow stars, respectively.

A phylogenetic tree was created based on the sequences of CsnB and other reported chitosanases from GH families 46, 75, and 80 (Figure 1). The phylogenetic tree indicates that the amino acid sequence of CsnB is highly similar with a GH family 46 chitosanase MH-Kl (Genbank number: BAA01474) from *Bacillus cirulans* MH-Kl. To further explore the structure of CsnB, a multiple sequence alignment was established among CsnB and other four chitosanases which is similar to CsnB, chitosanase MH-Kl, chitosanase from *Paenibacillus ehimensis* (Genbank number: BAA23489), and chitosanase from *Paenibacillus* sp. BH-2005 (Genbank number: ABC17783). Signal peptide analysis shows chitosanase MH-Kl (Genbank number: BAA01474) from *Bacillus cirulans*, which has the highest similarity with CsnB, has no signal peptide. The other two chitosanase have signal peptides founded in their N-termini. According to the multiple sequence alignment (Figure 2), the enzyme contains three conserved regions ‘TGLDGEQWNNIM’, ‘LFKAYDAAKGA’, and ‘PPNGKNRVKQW’. The regions are related to substrate binding and catalytic activity in GH family 46. Moreover, the conserved sites Tyr113 and Thr134 play key roles in the substrate preferences while the sites Glu103 and Asp121 are related to the hydrolysis catalyzing process.

### 2.2. Expression, Purification, and Characterization of csnB

**Figure 3:**
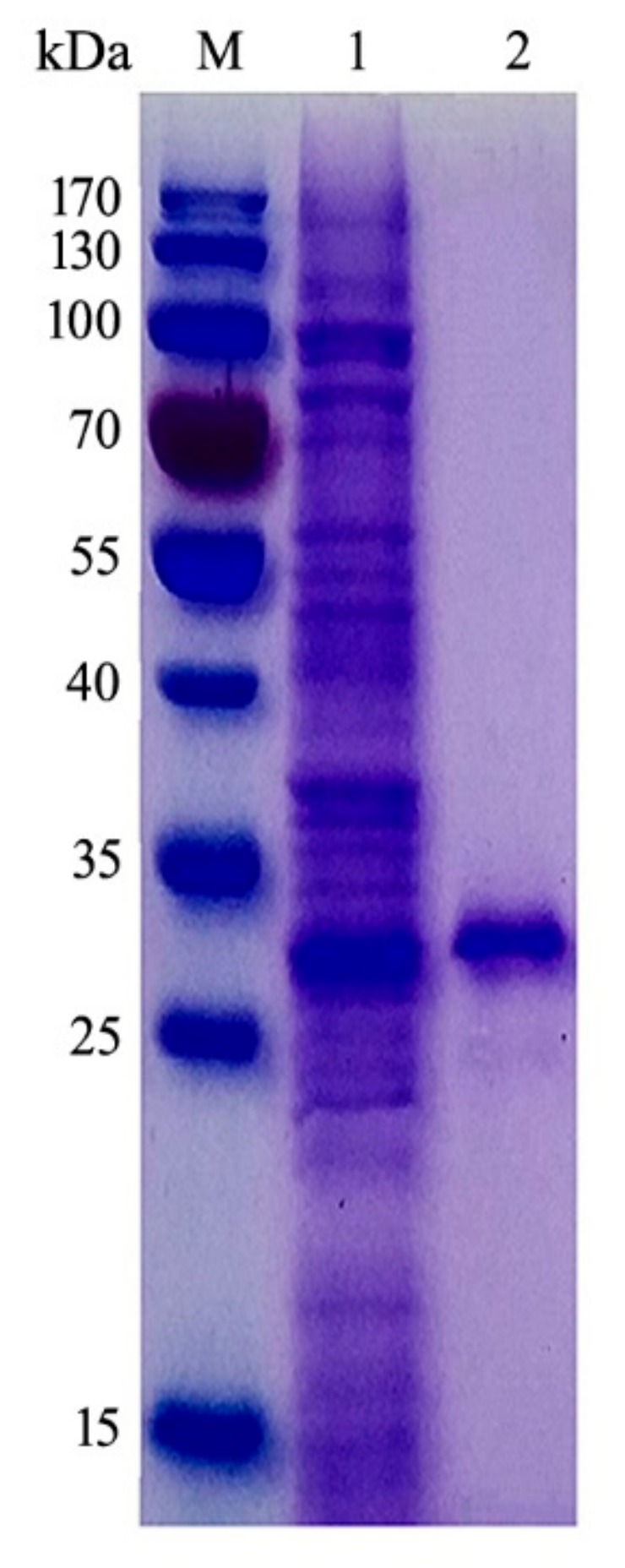
Dodecyl sulfate-polyacrylamide gel electrophoresis (SDS-PAGE) analysis of purified CsnB. M, molecular marker; 1, crude enzymes; 2, purified CsnB. The molecular weight of purified CsnB (theoretical molecular: 30.89 kDa) after Ni-NTA sepharose column was measured to be about 30 kDa by SDS-PAGE.

The *csnB* gene was expressed in the pET22b (+)/*E. coli* BL21 (DE3) system. Most of the recombinant proteins existed in a soluble state when the cells were induced at 20 °C for 20 h proving CsnB an extracellular secreted protein. The recombinant CsnB protein was purified to homogeneity with a specific activity of 329.3 U/mg. About 68.7 mg of purified protein was obtained from 1 L of bacterial culture using Ni-affinity chromatography. The Mw of purified CsnB was estimated to be about 30 kDa by dodecyl sulfate-polyacrylamide gel electrophoresis (SDS-PAGE) (Figure 3), which corresponded to the theoretical molecular mass of 30.89 kDa.

**Figure 4:**
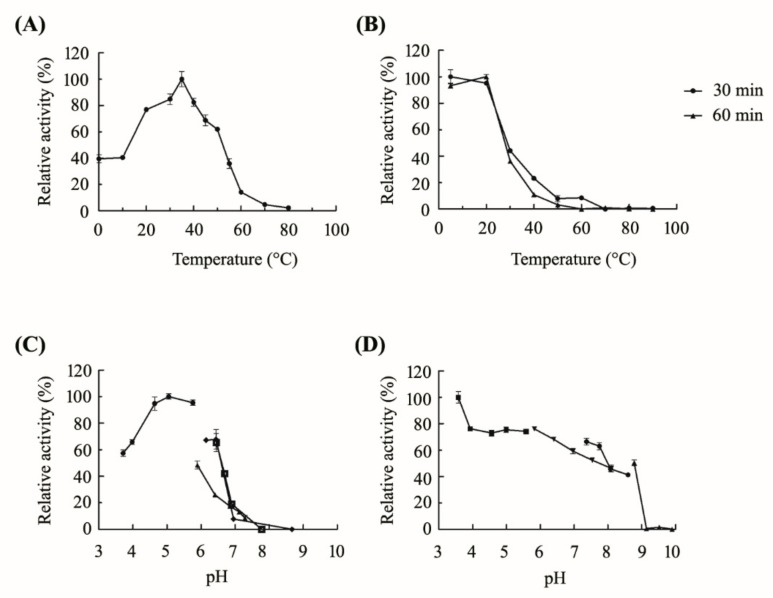
Effect of temperature and pH on CsnB activity. (**A**) Effect of temperature on the activity of chitosanase. (**B**) Thermo-stability of CsnB. Two groups of enzymes were incubated for 30 min or 60 min. (**C**) Effect of pH on the activity of chitosanase. (HAC-NaAC buffer, pH 3.71-5.76; phosphate buffer, pH 5.89-7.29; Tris-HCl buffer, pH 6.45-7.78; glycine-NaOH buffer, pH 6.15-8.67.) (**D**) pH-stability of CsnB. The pH-stability was analyzed by measuring the residual activity after pretreating the enzyme in different buffers at 4 °C for 24 h (HAC-NaAC buffer, pH 3.58-5.58; phosphate buffer, pH 6.97–8.10; Tris-HCl buffer, pH 7.37–8.60; glycine-NaOH buffer, pH 8.78-9.90). Values are the means of three independent experiments ± standard deviations.

Then, the purified CsnB was used to determine its biochemical properties. CsnB showed its maximum activity at 35 °C (Figure 4A). It was found that it maintained over 70% activity at 20 °C. Meanwhile, CsnB showed 39.5% and 40.4% of its maximum activity at 0 and 10 °C, respectively. (Figure 4A). This result indicated that this enzyme is a cold-adapted enzyme, which exhibited high activity at low temperature. Thus far, only a few cold-adapted chitosanase have been reported. Compared with the other three cold-tolerant or cold-adapted chitosanases previously reported, the residual activity of CsnB is much higher at 0–30 °C [5,20,21]. Cold-adapted CsnM from *Pseudoalteromonas* sp. SY39 showed 24.1%, 30.6%, and 49.4% of its maximum activity at 0, 10, and 15 °C [20], while CsnB maintained 39.5%, 40.4%, and 76.8% of its maximum activity at 0, 10, and 20 °C, respectively. In addition, the optimal temperature of cold-adapted chitosanase from *Janthinobacterium* sp. 4239 was 45 °C and maintained its 30%–60% activity at 10–20 °C [5]. Another cold-adapted enzyme, GsCsn46A from *Gynuella sunshinyii* was reported to maintain 70% of its maximum activity at 15 °C [21]. Overall, CsnB showed its irreplaceable advantages over these chitosanases. Cold-adapted enzymes can perform biocatalytic processes at low temperatures and control the hydrolysis of substrates under mild reaction conditions. Furthermore, low temperature could inhibit bacterial growth and increase the purity of products. Therefore, the application of cold-adapted CsnB in the industrial production has become an interesting research area.

To measure the thermo-stability of CsnB, the enzyme was incubated in different temperatures for pre-treatment for 30 and 60 min, respectively. Then, the residual activity was measured at its optimum temperature and pH. As it showed in Figure 4B, residual activity was 95% of its maximum activity after incubation for 30 min at 20 °C. While, the residual activities have been recorded a steady decrease to a rather lower level as temperature increase higher. The optimum pH for CsnB was determined to be 5.0 in sodium acetate buffer (Figure 4C). And it was found that it maintained relatively high activity in the pH 4.64–5.76. Meanwhile, it maintained 57.4% and 65.8% of its maximum activity in pH 3.71 and pH 3.98 of acetic acid-sodium acetate (HAC-NaAC), respectively, indicating a good adaption of acid environment (Figure 4C). CsnB exhibited wide pH-stability within the range of pH 3.58~5.58 of HAC-NaAC retaining over 70% of its initial activity (Figure 4D). Meanwhile, CsnB performed remarkably stability with pH declining, Therefore, CsnB could be used in a strong acidic environment.

The effects of metal ions and organic reagents on the activity of CsnB were shown in Table 1. Among all the metal ions tested, Mn^2+^, Cu^2+^, Mg^2+^ and Li^+^ obviously enhanced enzymatic activity, wherein Mn^2+^ increased enzyme activity to 257%. Additionally, NH_4_^+^, Ba^2+^, Ca^2+^ and K^+^ slightly increased the activity of CsnB. Surprisingly, the metal ion Cu^2+^ could strongly inhibit the activity of the family 75 chitosanase from *Aspergillus* sp. W-2 activity over 96% at both 1 and 10 mM [16], but the activity of CsnB increased to 136%. The GH-46 family chitosanases reported previously shows the metal ion binding sites, and these metal ions may bind to these sites, thereby enhancing the three-dimensional structural stability of chitosanases. Adversely, CsnB was significantly inhibited in the presence of EDTA, which further indicated that the bivalent metal ions play an important role in enzyme activity. Moreover, activities of CsnB were enhanced by NaCl (concentrations vary from 5 to 500 mM) and reached its maximum activity at 250 mM, at which point the activity was about 1.5 times higher than the activity in the absence of NaCl (Table 1).

### 2.3. Effect of Glycerol on the Thermo-Stability of CsnB

**Figure 5:**
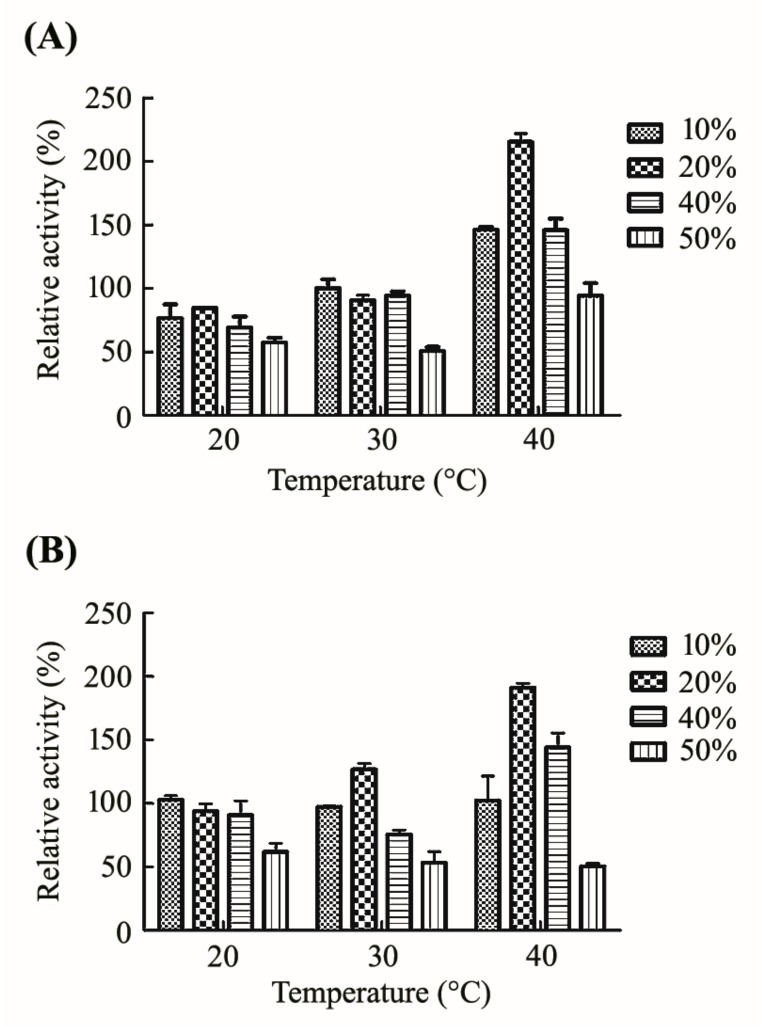
The effect of glycerol on the thermo-stability (20, 30, 40 °C) of CsnB. (**A**) Residual activity after pretreatment for 30 min. (**B**) Residual activity after pretreatment for 60 min. The activities of CsnB without glycerol at the same temperature were defined as the control at 100%.

It is a normal phenomenon that cold-adapted enzymes are unstable when the reaction temperatures increase [19]. In this study, we have discovered a method to improve its stability by adding different concentrations of glycerol. As a result, the thermo-stability of CsnB was improved. As shown in Figure 5A, when the enzyme was pre-treated at 20 °C and 30 °C, the positive effect of glycerol was not obvious. The residual activities maintained 76.5%, 84.6%, 69.2%, and 57.5% after 30 min incubation at 20 °C with a final glycerol concentration of 10%, 20%, 40%, and 50%. It is noteworthy that glycerol of any concentration had no positive effect on the activity of CsnB at 20 °C. When the reaction temperature increased to 30 °C, the effects slightly increased. The activity of CsnB was increased by glycerol when the temperature reached 40 °C. After incubation at 40 °C for 30 min, the activity of CsnB with 20% glycerol increased to 215.3% dramatically. The activities also had an improvement to various degrees when the glycerol concentration was 10% and 40%. However, the mixture with 50% glycerol showed adverse effects at 20, 30, and 40 °C, which may be attributed to an exaggerate concentration of glycerol. The effects were similar after pretreatment for 60 min (Figure 5B). We predicted that glycerol acted as an enzymatic protective reagent according to the results of the experiment, improving the temperature stability of CsnB. It would show the opposite effect when the temperature is low. It was also worth mentioning that the normal reaction was inhibited when the glycerol solution was diluted or at a too high concentration. Together, CHOS production at room temperature without precise temperature control could be achieved using this method, which suggests that the cold-adapted property deficiency of CsnB could be avoided and that potential advantages were exhibited in large-scale industrial applications.

Glycerol could improve the stability of enzymes is a common phenomenon. In the presence of glycerol, the fluctuation of protein molecules decreases [22]. The protective mechanism of polyols on protein still remains debatable. Preferential hydration has been the dominated mechanism to elucidate this protecting effect. Specifically, the exclusion of glycerol from protein surface leads to the favorable interaction between the water and protein molecules [23]. A previous study about chitosanase N174 also mentioned the protective effect of glycerol [24], while suggesting that glycerol’s effects on it are not as obvious as on CsnB.

### 2.4. Action Mode and Reaction Products

**Figure 6:**
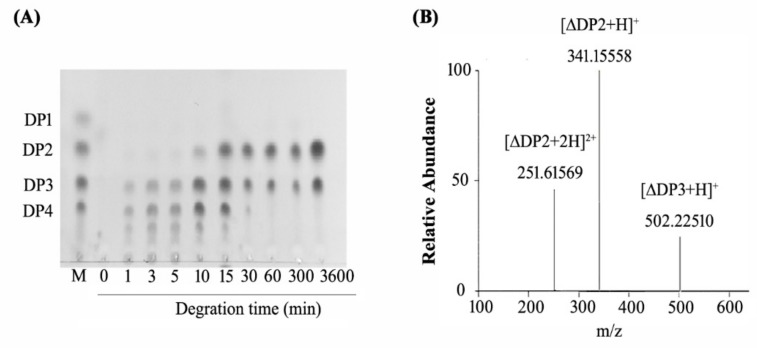
TLC and ESI-MS analysis of enzymatic hydrolytic products. (**A**) TLC analysis of productions after different reaction times. Lane M: standard chitooligomers (DP = 1–4). (**B**) ESI-MS analysis. (Disaccharindes: 251.61569 *m*/*z*, [∆DP2 + 2H]^2+^; 341.15558 *m*/*z*, [∆DP2 + H]^+^. Trisaccharides: 502.22510 *m*/*z*, [∆DP3 + H]^+^.).

The action mode of CsnB was analyzed by thin-layer chromatography (TLC) and positive-ion electrospray ionization mass spectrometry (ESI-MS) (Figure 6). As shown in TLC analysis (Figure 6A), CHOS with different degree of polysaccharides (DPs) appeared after degrading for 1–5 min. With the reaction time being prolonged, the relative proportions of DP2 and DP3 oligomers increased while the higher DP products decreased. Only two visible spots were left on the TLC plate after complete degradation. The mobility ratios of the spots were identical by the chitodimer and chitotrimer markers. Hence, the end products of CsnB were proven to be disaccharides and trisaccharides. Monosaccharide was not detected. Therefore, CsnB is an endo-type enzyme catalyzing the cleavage of β-1,4-glycosidic linkage specifically.

To further investigate the composition and DP of the end products, a positive-ion ESI-MS was employed. As shown in Figure 6B, three main signal peaks 251.61569 *m*/*z*, 341.15558 *m*/*z*, and 502.22510 *m*/*z* correspond to [∆DP2 + 2H]^2+^, [∆DP2 + H]^+^ and [∆DP3 + H]^+^ respectively, which corresponding to the molecular weight of chitosan disaccharindes (DP2) and trisaccharides (DP3). These results indicated that the final products of CsnB are chitosan disaccharindes and trisaccharides.

## 3. Materials and Methods

### 3.1. Materials

The expression vector, pET22b (+), were purchased from Takara, Dalian, China. The *E. coli* strains BL21 (DE3) was purchased from Novagen, USA. Chitosan (degree of deacetylation ≥ 95%, viscosity: 100–200 mpa.s) was purchased from Aladdin Biochemical Technology Co., Ltd. (Shanghai, China). BCA protein assay kit was purchased from Beyotime Biotechnology, Shanghai, China. The thin-layer chromatography (TLC) silica gel plates were 60 F254 from Merck KGaA, 64,271 Darmstadt, Germany. Mass Spectrometer (ThermoFisher Q Exactive Hybrid Quadrupole-Orbitrap, German) was utilized for ESI-MS analysis.

### 3.2. Isolation of the Bacterial Strains

The chitosanase-producing bacterial strains were isolated from the sediment surface layer of Yellow Sea bottom (depth 36 m, E 120.13, N 35.76, collected in May, 2017), The isolation medium contains 0.50% chitosan, 0.10% KH_2_PO_4_, 0.20% K_2_HPO_4_, 0.07%MgSO_4_, 0.10% NaCl, and 0.01% CaCl_2_ and 1% agar. Several colonies were selected as being positive. Then the most active strain was picked out for 16S rRNA gene sequence analysis. 

### 3.3. Sequence Analysis of csnB/CsnB

The genomic sequence analysis of *Bacillus* sp. BY01 indicated that it contained a chitosanase-encoding gene, named *csnB*. The gene was chosen for the gene cloning and chitosanase expression. The sequence cloned from the genome of *Bacillus* sp. BY01 was deposited at Genbank database (accession number: MN531545). To further analyze the sequence, the ORF was identified using the program ORF finder (https://www.ncbi.nlm.nih.gov/orffinder/). Signal peptides of amino-acid sequences were analyzed using the SignalP 5.0 server (http://www.cbs.dtu.dk/services/SignalP/). To refine the phylogenetic analysis, Conserved Domain Database (CDD) was used to obtain its domain and family information. Subsequently, searches of the similar sequences of *csnB* were analyzed with the BLAST algorithm program on the National Center for Biotechnology Information (NCBI) server (https://www.ncbi.nlm.nih.gov/). Multiple sequence alignment was carried out using ClustalX2.1 and ESPript (http://espript.ibcp.fr/ESPript/cgi-bin/ESPript.cgi). Then the phylogenetic tree was performed via the bootstrapping neighbor-joining method using MEGA7.0. In addition, ExPASy (https://web.expasy.org/cgi-bin/protparam/protparam) was used to determine the theoretical isoelectric point (pI) and molecular weight (Mw) of CsnB.

### 3.4. Expression of Recombinant csnB

The *csnB* fragment with signal peptide and terminator was amplified using polymerase chain reaction (PCR). The PCR primers were Ep-*csnB*-F and Ep-*csnB*-R (Table S1), which contained two recognition sites *Nde* I and *Xho* I respectively. The amplified sequence was inserted into plasmid pET22b between the same recognition sites. The *E. coli* BL21 (DE3) with recombinant plasmid pET22b-*csnB* plasmid were grown in Luria-Bertani (LB) broth for 6 h at 37 °C for proliferation. Afterwards, *E. coli* BL21-pET22b-*csnB* cells were cultured in TB medium contained 50 μg/mL ampicillin at 37 °C for CsnB expression. When OD600 reached 0.6–0.8, isopropyl β-d-thiogalactoside (IPTG, 0.1 mM) was added in order to induce protein expression then the *E. coli* BL21-pET22b-*csnB* were transferred to 20 °C for 96 h. 

### 3.5. Purification and Characterization of csnB

After centrifuging the cultures at 4 °C with 9000 rpm for 20 min, the supernatant was loaded onto a Ni-NTA sepharose column (GE Healthcare, Little Chalfont, Buckinghamshire, UK) and performed purification process with AKTA150 automatic purification system. Imidazole of gradient concentration (25–500 mM) in 20 mM phosphate buffer (pH 7.6, 500 mM NaCl) was used for the elution of target protein. The molecular weight and purity of the purified CsnB were assayed by SDS-PAGE on a 12% (*w*/*v*) resolving gel was utilized to measure the concentration of CsnB using bovine serum albumin (BSA) as standard. The optimal temperature of CsnB was determined at different temperatures (0–80 °C). To measure the thermo-stability of CsnB, the purified CsnB was firstly incubated at various temperatures (0–100 °C) for 30 min and 60 min, respectively. Then, the enzymatic activity was determined at its optimal condition. To determine its optimal pH, the enzyme was fixed with different buffer as follows: HAC-NaAC, pH 3.71–5.76; phosphate buffer, pH 5.89–7.29; Tris-HCl buffer, pH 6.45–7.78; and glycine-NaOH buffer, pH 6.15–8.67. Moreover, the pH-stability of CsnB was determined at its optimal condition after the enzymes incubated at different buffers [HAC-NaAC (pH 3.58–5.58); phosphate buffer (pH 6.97-8.10); Tris-HCl buffer (pH 7.37–8.60); glycine-NaOH buffer (pH 8.78–9.90)] at 4 °C for 24 h.

### 3.6. Chitosanase Activity Assay

The activity of chitosanase was determined by 3,5-dinitrosalicylic acid (DNS) method [25,26] Briefly, 450 μL chitosan substrate [0.3% (*w*/*v*), pH 5] was pre-incubated at 35 °C for 10 min. Chitosan substrate was prepared with chitosan powder (3 g) and distilled water. NaOH and HCl were used to adjust pH to 5, and the final volume was fixed to 1000mL then sterilized. Enzyme was diluted to a proper concentration to make sure the activity measured was in the optimal range of the colorimetric method. Then, 50 μL diluted enzyme was mixed with the substrate and the reaction mixture was incubated at 35 °C for 10 min. Then, 375 μL of DNS was added to the reaction mixture to terminate the reaction. After this, we boiled the mixture for 10 min to change its color. The mixture was centrifuged at 12,000 rpm for 4 min to remove the precipitate. The reducing sugar in the supernatant was calculated by the colorimetric method. One unit of chitosanase activity was defined as the amount of enzyme that liberated 1 μmol of reducing sugar per min under the optimal conditions.

### 3.7. Effect of Glycerol on the Thermo-Stability of csnB

Glycerol was added to purified CsnB to improve the thermo-stability of CsnB. The final concentrations of glycerol were made to be 10%–50%. To further evaluate the effect of glycerol on the thermo-stability, the residual activity was measured after incubation at different temperatures (20, 30, 40 °C) for 30 min or 60 min. The activities of CsnB without glycerol at the same temperature were defined as the control at 100%.

### 3.8. Effects of Metal Ions and Organic Reagents on csnB

To determine the effect of metal ions and other organic reagents on CsnB activity, the activity of purified CsnB was assayed after the addition of Mg^2+^, Cu^2+^, Ca^2+^, Mn^2+^, Ba^2+^, Co^2+^, SDS, EDTA, K^+^, Li^+^, NH_4_^+^, Fe^3+^, and Al^3+^ to the reaction mixtures at a final concentration of 5 mM. Additionally, we explored the effect of different concentrations (0, 5, 25, 50, 100, 250, and 500 mM) of NaCl on the activity of CsnB. The activity assays were determined under the optimum conditions of CsnB and the activity of CsnB assayed without adding metal ions or organic reagents was taken as the control and defined as 100%.

### 3.9. Degradation Production Analysis

To analyze the enzymatic products, TLC method was utilized. CsnB (3 mL) was added into chitosan solution (0.3%,15 mL) and solution (500 µL) was picked out and boiled after different reaction times (0, 1, 3, 5, 10, 15, 30, 60, 300, and 3600 min). Samples (2.5 µL) were added onto the TLC plate and put into the developing solvent. Standard chitosan oligomer mixture used in this study were prepared in our lab. The developing solvent was a mixture of ammonia, water, and isopropanol in volume ratios of 3:27:70. Then 0.5% ninhydrin ethanol solution was sprayed onto the TLC plate after drying and the plate was heated at 80 °C for 30 min for final visualization. To clarify the components and degree of polymerization (DP) of the final products, positive-ion electrospray ionization mass spectrometry (ESI-MS) was subsequently used.

## 4. Conclusions

In this study, we cloned and characterized a new GH46 chitosanase, CsnB, from the marine bacterium *Bacillus* sp. BY01. Our study demonstrated that the combined properties of CsnB, such as cold-adaption, wide pH-stability, and high CHOS yield make it an excellent candidate for further study and adaptation for commercial use. Further analyses will focus on elucidating the molecular mechanism of CsnB, along with the determination of its three-dimensional structure.

## Figures and Tables

**Table 1:** Effects of metal ions, EDTA and SDS on the activity of CsnB.

Reagent Added	Concentration (mM)	Relative Activity (%)
None	-	100 ± 2.7
NaCl	5	106 ± 9.6
	25	118 ± 4.6
	50	118 ± 8.0
	100	125 ± 11.8
	250	151 ± 1.1
	500	145 ± 5.7
MnCl_2_	5	257 ± 5.1
CuSO_4_	5	136 ± 7.6
MgSO_4_	5	132 ± 5.5
Li_2_SO_4_	5	130 ± 7.6
NH_4_Cl	5	115 ± 5.9
BaCl_2_	5	111 ± 7.7
CaCl_2_	5	109 ± 6.6
KCl	5	103 ± 10.1
CoCl_2_	5	89 ± 3.6
SDS	5	83 ± 5.7
Al_2_(SO_4_)_3_	5	81 ± 9.6
FeCl_3_	5	4 ± 2.2
EDTA	5	0 ± 0.0

Activity without addition of chemicals was defined as 100%. Data are shown as means ± SD.

## Supplementary Materials

The following are available online, Table S1: Primers used in this study.
