# Supplementary material for: Cloning and Characterization of a Cold-adapted Chitosanase from Marine Bacterium Bacillus sp. BY01
Source: Molecules. 2019 Oct 30;24(21):3915. doi: 10.3390/molecules24213915 (PMC6864755; doi:10.3390/molecules24213915)
Supplement: Supplementary file 1 [file molecules-24-03915-s001.pdf]

Table S1 Primers used in this study

Primers	sequences
Ep-csnB-F	5′- GGGAATTC CATATGATGAAATACCTGCTGCCGAC-3′
Ep-csnB-F	5′- CCG CTCGAG ATCCCACTGTTTCACACG-3′
